# Supplementary material for: Dietary Oyster (Crassostrea gigas) Extract Ameliorates Dextran Sulfate Sodium-Induced Chronic Experimental Colitis by Improving the Composition of Gut Microbiota in Mice
Source: Foods. 2022 Jul 8;11(14):2032. doi: 10.3390/foods11142032 (PMC9317888; doi:10.3390/foods11142032)
Supplement: Supplementary file 1 [file foods-11-02032-s001.zip › foods-1771485-supplementary.pdf]

Supplementary Materials

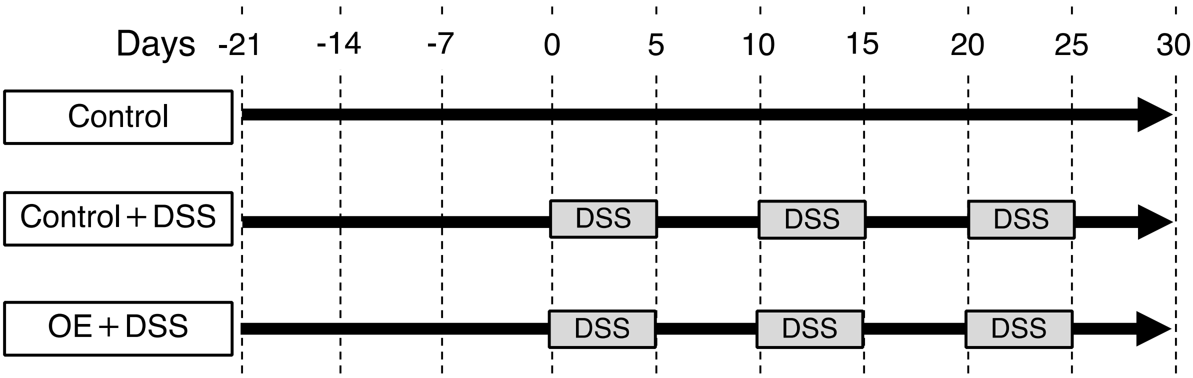

Figure S1. Schematic diagram illustrating the induction of chronic experimental colitis symptoms. DSS, dextran sulfate sodium; OE, oyster extract.

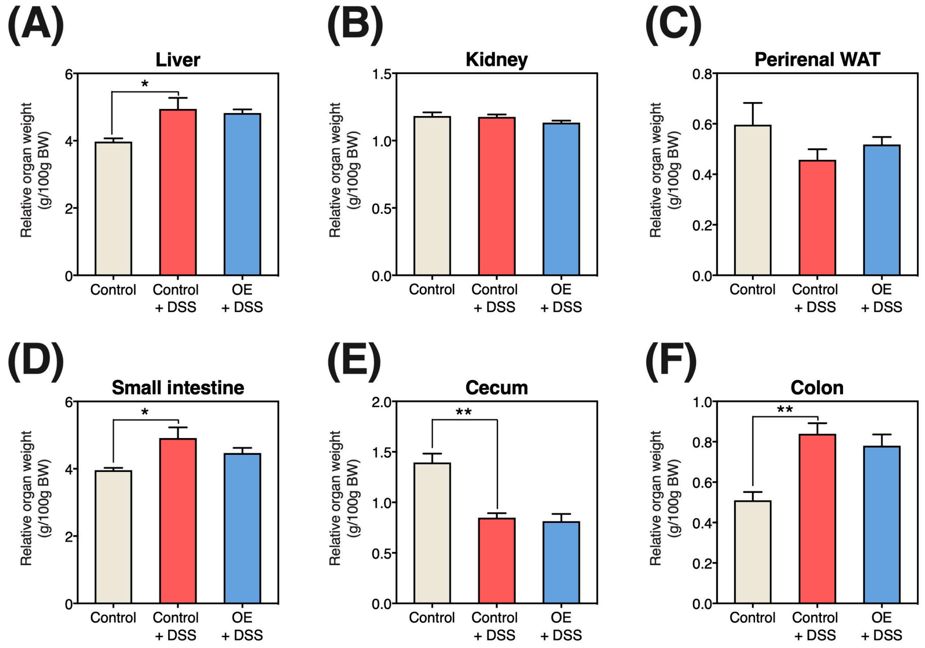

Figure S2. Relative weights of organs. Relative weights of **(A)** liver, **(B)** kidney, **(C)** perirenal white adipose tissue (WAT), **(D)** small intestine, **(E)** cecum, and **(F)** colon on day 30 of dextran sulfate sodium (DSS) administration. The weights of the small intestine, cecum, and colon includes their contents. Results are presented as mean ± standard error of the mean (n = 6 for the control group and n = 8 for groups the control + DSS and oyster extract (OE) + DSS groups). * $p < 0.05$ and ** $p < 0.01$. OE, oyster extract.

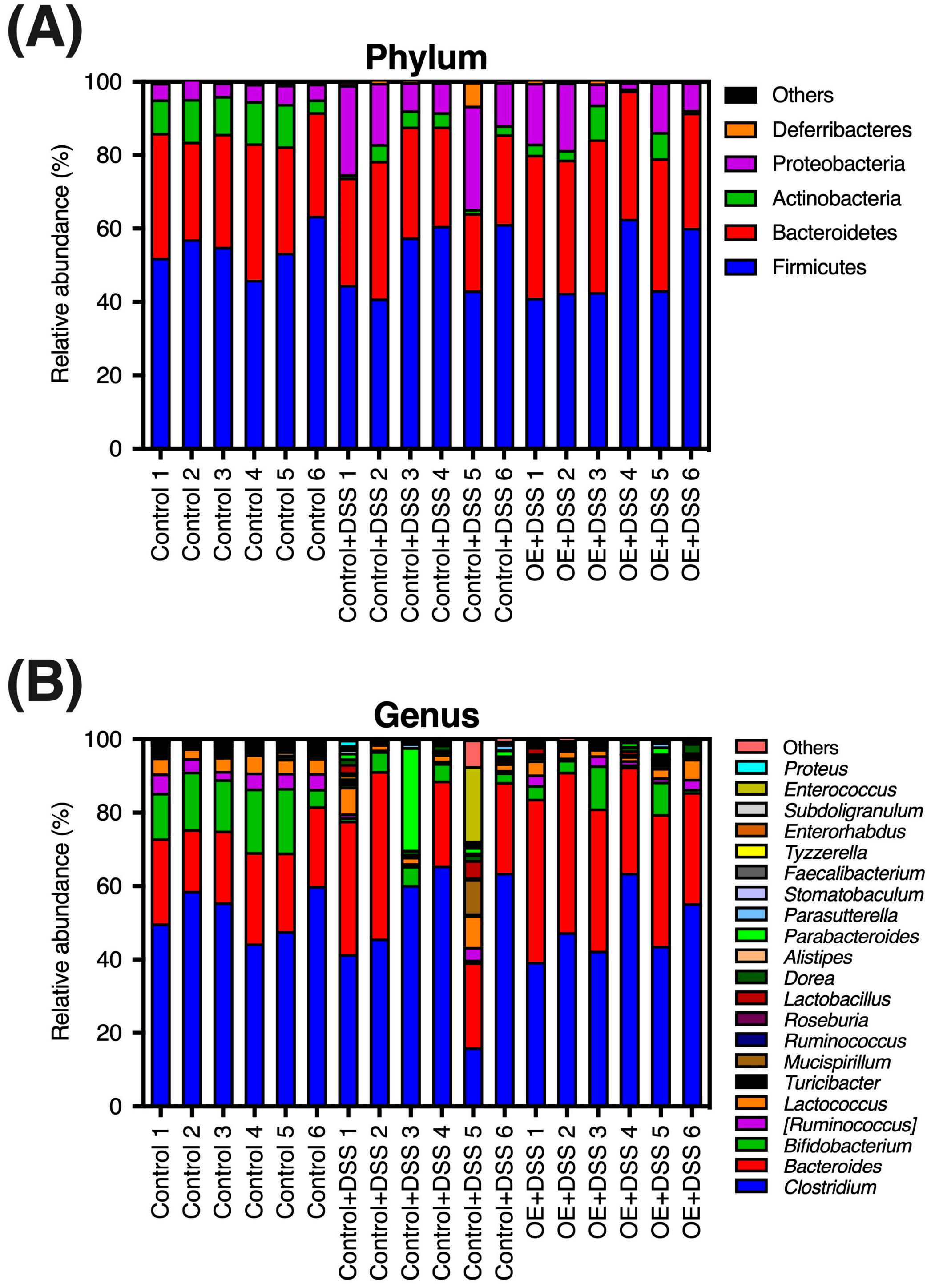

Figure S3. Relative abundance of fecal bacterial phyla and genera. **(A)** Relative fecal bacterial abundance at the phylum level. **(B)** Relative fecal bacterial abundance at the genus level. The relative fecal bacteria abundance was sorted from the highest relative abundance in the control group, and those with a relative abundance of less than 0.5% were grouped as "others". DSS, dextran sulfate sodium; OE, oyster extract.

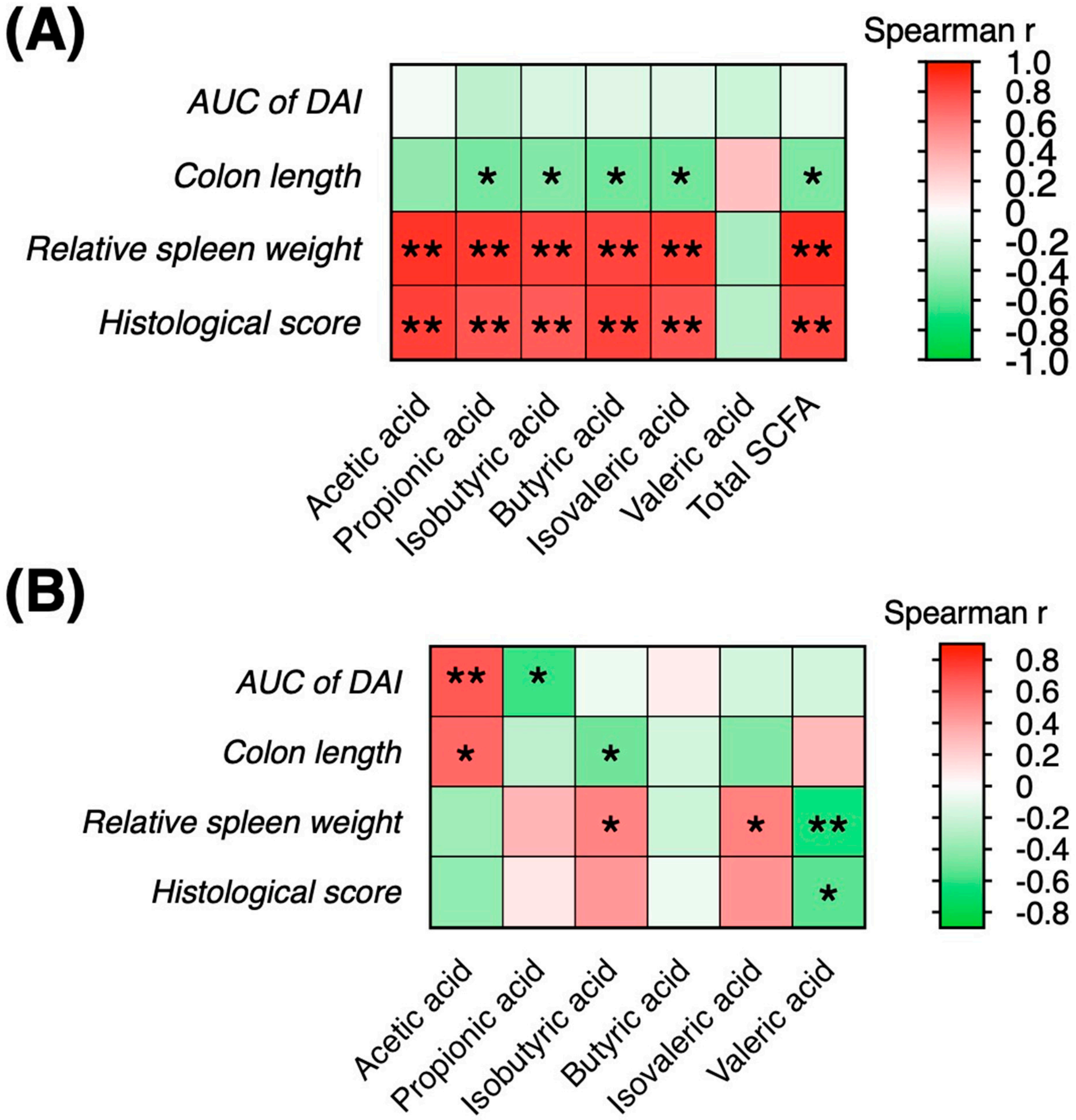

Figure S4. Heatmap with Spearman's correlation coefficient between indicators of chronic experimental colitis severity and fecal short-chain fatty acids (SCFA). **(A)** Indicators of chronic experimental colitis severity induced by dextran sulfate sodium (DSS) and fecal SCFA contents. **(B)** Indicators of chronic experimental colitis severity induced by DSS and relative content of each SCFA. Red: positive correlations; green: negative correlations. * $p < 0.05$ and ** $p < 0.01$. AUC, area under the curve; DAI, disease activity index.

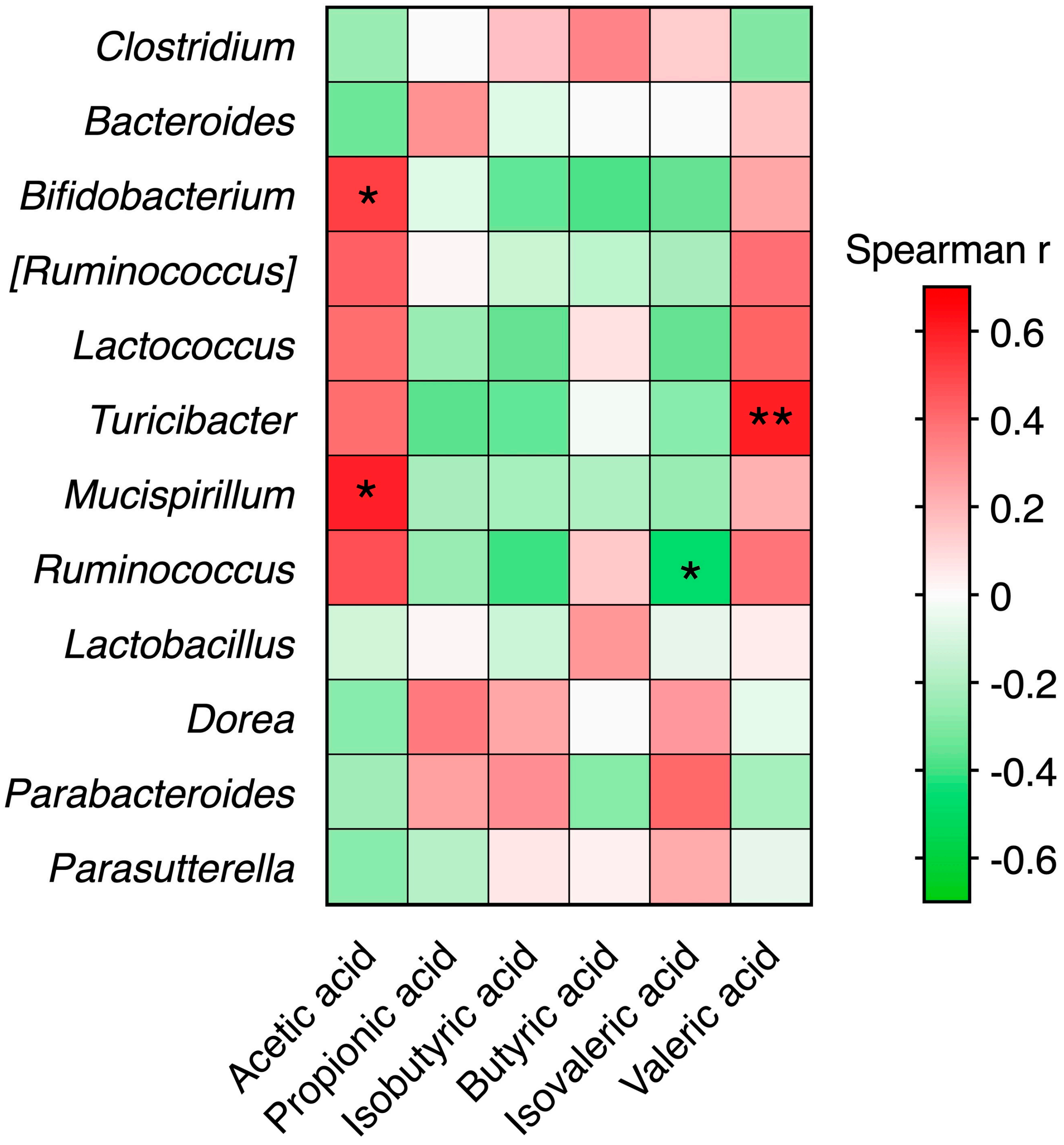

Figure S5. Heatmap with Spearman's correlation coefficient between the relative content of each short-chain fatty acids (SCFA) and bacteria in feces. Red: positive correlations; green: negative correlations. * $p < 0.05$ and ** $p < 0.01$.

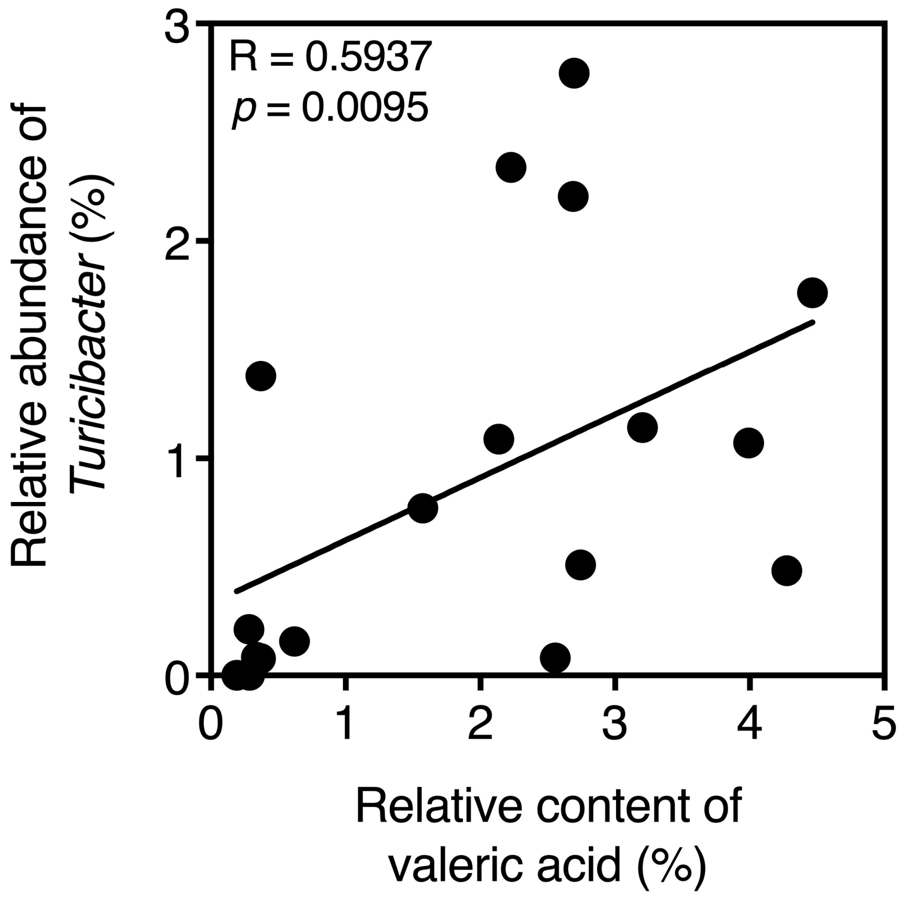

Figure S6. Relationship between the relative fecal content of valeric acid and relative abundance of *Turicibacter*.

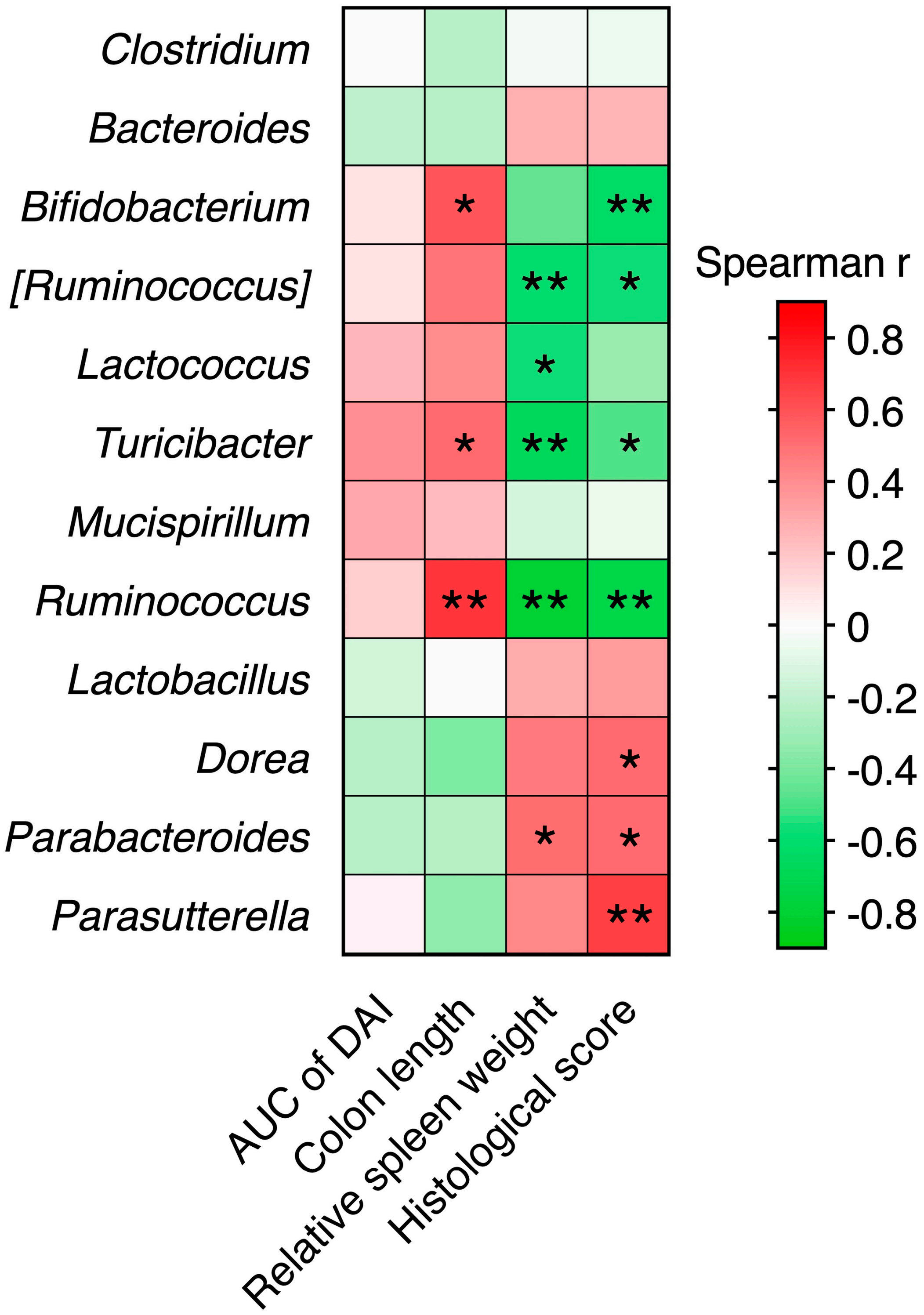

Figure S7. Heatmap with Spearman's correlation coefficient between the indicators of chronic experimental colitis severity and relative abundance of bacteria in feces. Red: positive correlations; green: negative correlations. * $p < 0.05$ and ** $p < 0.01$. AUC, area under the curve; DAI, disease activity index.

Table S1. Experimental diet composition.

	Experimental diets[1]	
	Control	OE
Casein	200	182.3
Dextrinized corn starch	132	132
Corn starch	397.486	366.486
Sucrose	98.47	100
Cellulose	50	50
L-Cystine	3	3
Choline bitartrate	2.5	2.5
AIN-93G mineral mixture	35	
Sodium chloride-free AIN-93G mineral mixture		35
Sodium chloride	1.53	
AIN-93 vitamin mixture	10	10
Soybean oil	70	68.7
tert-Butylhydroquinone	0.014	0.014
OE		50

[1]Based on the AIN-93G formula. The amounts of sodium chloride in the control and OE diets were equal. The diet ingredients required to prepare AIN-93G were purchased from Fujifilm Wako Pure Chemical Co. (Osaka, Japan) and Oriental Yeast Co., Ltd. (Tokyo, Japan). AIN, American Institute of Nutrition; OE, oyster extract.

Table S2. Growth parameters.

	Experimental groups		
	Control	Control + DSS	OE + DSS
Growth parameters			
Food intake (g/day)	2.95 ± 0.02	2.93 ± 0.07	2.87 ± 0.03
Water intake (g/day)	5.82 ± 0.13	6.58 ± 0.13	6.62 ± 0.10
DSS solution intake (g/day)	-	7.33 ± 0.14	7.24 ± 0.10

Results are presented as mean ± standard error of the mean (n = 6 for the control group and n = 8 for the control + DSS and OE + DSS groups). DSS, dextran sulfate sodium; OE, oyster extract.

Table S3. Serum biochemical parameters.

	Experimental groups		
	Control	Control + DSS	OE + DSS
Total protein (g/dL)	4.42 ± 0.08	4.40 ± 0.10	4.31 ± 0.12
Albumin (g/dL)	2.43 ± 0.14	2.40 ± 0.08	2.30 ± 0.11
Alanine aminotransferase (IU/L)	36.8 ± 1.3	38.4 ± 2.0	37.1 ± 1.3
Aspartate aminotransferase (IU/L)	10.0 ± 0.9	11.9 ± 0.7	11.0 ± 0.7
Creatine phosphokinase (IU/L)	32.2 ± 4.5	38.5 ± 4.7	31.8 ± 1.9
Lactate dehydrogenase (IU/L)	187 ± 5	187 ± 4	171 ± 11
Urea nitrogen (mg/dL)	28.2 ± 1.0	29.0 ± 1.2	27.5 ± 1.8
Creatinine (mg/dL)	0.14 ± 0.01	0.13 ± 0.01	0.14 ± 0.01
Triglyceride (mg/dL)	41.7 ± 7.6	58.3 ± 7.3	61.4 ± 11.6
Total cholesterol (mg/dL)	117 ± 4	114 ± 4	110 ± 4
High-density lipoprotein cholesterol (mg/dL)	93.3 ± 1.8	87.9 ± 3.7	84.3 ± 2.8
Phospholipids (mg/dL)	266 ± 6	267 ± 6	249 ± 6

Results are presented as mean ± standard error of the mean (n = 6 for the control group and n = 8 for the control + DSS and OE + DSS groups). DSS, dextran sulfate sodium; OE, oyster extract.
